# TRAIL-coated leukocytes to kill circulating tumor cells in the flowing blood from prostate cancer patients

**DOI:** 10.1186/s12885-021-08589-8

**Published:** 2021-08-06

**Authors:** Nerymar Ortiz-Otero, Jocelyn R. Marshall, Antonio Glenn, Jubin Matloubieh, Jean Joseph, Deepak M. Sahasrabudhe, Edward M. Messing, Michael R. King

**Affiliations:** 1 Meinig School of Biomedical Engineering, Cornell University, Ithaca, NY 14850 USA; 2 Department of Biomedical Engineering, Vanderbilt University, Nashville, TN 37202 USA; 3 The University of Rochester Medical Center, Rochester, NY 14642 USA

**Keywords:** Prostatectomy, CTC mobilization, CAFs, CTC cluster, Cancer recurrence, TRAIL-liposomal therapy

## Abstract

**Background:**

Radical surgery is the first line treatment for localized prostate cancer (PC), however, several studies have demonstrated that surgical procedures induce tumor cell mobilization from the primary tumor into the bloodstream.

**Methods:**

The number and temporal fluctuations of circulating tumor cells (CTC), cancer associated fibroblasts (CAF) and CTC cluster present in each blood sample was determined.

**Results:**

The results show that both CTC and CTC cluster levels significantly increased immediately following primary tumor resection, but returned to baseline within 2 weeks post-surgery. In contrast, the CAF level decreased over time. In patients who experienced PC recurrence within months after resection, CTC, CAF, and cluster levels all increased over time. Based on this observation, we tested the efficacy of an experimental TNF-related apoptosis-inducing ligand (TRAIL)-based liposomal therapy ex-vivo to induce apoptosis in CTC in blood. The TRAIL-based therapy killed approximately 75% of single CTCs and CTC in cluster form.

**Conclusion:**

Collectively, these data indicate that CTC cluster and CAF levels can be used as a predictive biomarker for cancer recurrence. Moreover, for the first time, we demonstrate the efficacy of our TRAIL-based liposomal therapy to target and kill prostate CTC in primary patient blood samples, suggesting a potential new adjuvant therapy to use in combination with surgery.

**Supplementary Information:**

The online version contains supplementary material available at 10.1186/s12885-021-08589-8.

## Background

Prostate cancer (PC) is the second most frequent cancer diagnosed in men worldwide. Although the estimated 5-year survival rate of PC is approximately 98.9%, cancer recurrence, metastatic progression, and the inevitable emergence of resistance to hormonal therapy makes PC the second leading cause of cancer death in men in western countries and the fifth leading cause of cancer-related death worldwide [1–3]. For localized PC, a first line treatment is radical prostatectomy (RP) followed, in some cases, by radiotherapy (XRT), as well as androgen deprivation therapy (ADT) [4].

Although these cancer treatments prolong patient life expectancy, ~ 50% of patients will experience cancer recurrence [5, 6]. Evidence has shown that prostatectomy and radiotherapy can induce CTC mobilization from the primary tumor site into the circulation, promoting tumor seeding in local and distant tissues due to the disruption of the tumor microenvironment [7–9]. Previous studies suggest that tumor cells may migrate from the primary tumor as CTC clusters containing up to 40 cells per cluster. These CTC clusters can contain a heterogeneous group of cells including immune cells, CAF, epithelial cells, and platelets. Moreover, CTC clusters are considered to have a 50–fold greater metastatic potential than single CTCs [10–13]. The higher metastatic potential in CTC clusters could be attributed to the presence of CAFs, which have been shown to promote tumor overgrowth in distant organs [14]. In PC, the tumor microenvironment is composed mostly of an accumulation of activated CAFs, whose main role is to promote tumor cell proliferation, invasion, angiogenesis, and epithelial-to-mesenchymal transition [15–18]. Recent studies have found that CAF level and number of CTC-CAF clusters in the circulation can predict cancer prognosis, with elevated levels correlating with worse prognoses in breast, colorectal cancer and PC [19, 20].

Due to the adverse consequences of CTC release after initial treatments, there is a need for treatments targeting CTCs in the bloodstream to reduce tumor recurrence in patients with PC. We previously developed a TRAIL-based therapy that targets and kills CTCs in the bloodstream. This therapy consists of liposomes decorated with E-selectin and TRAIL. E-selectin is a natural adhesion marker supporting endothelial cell and leukocyte rolling and adhesion, whereas TRAIL is a ligand that induces apoptosis in cancer cells while sparing normal cells. In in vitro studies, the therapy killed over 90% of PC cells under flow conditions during a treatment period of 2 h [21]. Similarly, in an orthotopic PC xenograph model, the TRAIL therapy killed over 90% of the CTCs and prevented the formation of macro-metastasis in distant organs [22, 23]. Based on these results, we hypothesized that the use of TRAIL therapy as a peri-operative treatment could kill primary CTCs in the blood of PC patients, under circulatory flow conditions.

In this study, we determined the fluctuations of single CTCs, CTC clusters and CAFs in primary blood samples from patients, and the correlation between these circulating cells and cancer recurrence. In addition, we demonstrated for the first time the use of TRAIL therapy to target and kill CTCs and CTC clusters in blood samples from PC patients under flow conditions.

## Methods

### Study approval

The study presented here was performed following the relevant guidelines and regulations (IRB#170222). To participate in this study, all of the PC patients and healthy volunteers received and signed a verbal and written consent form indicating the research study objectives. In addition, during the blood sample collection process, the identity of PC patients was completely protected.

### Blood sample collection

Samples of approximately 7.5 mL of peripheral whole blood were collected from PC patients using heparin coated tubes at four different time points during their cancer treatment: diagnosis, surgery, 1–2 days post-surgery and 1–2 weeks post-surgery. The blood collection procedure was initiated after the clinician diagnosed the PC patient via examination. The time period between the blood drawn (during treatment period) was similar between each patient. However, 4 samples were excluded in this study due to patient withdrawal. The blood samples were shipped from the University of Rochester Medical Center to Vanderbilt University overnight at ambient temperature and were then split into 3 aliquots to be treated as: untreated sample (UNT), vehicle control, and TRAIL liposomal therapy. The blood was maintained in a rotator before processing to avoid blood coagulation.

### CTC and CAF isolation from PC patient blood samples

Buffy coat was isolated from blood aliquots (UNT) using Ficoll by density gradient described before [24]. CTCs within the buffy coat were isolated using a negative selection kit with CD45 magnetic beads (Mylteni Biotech). The buffy coat pellet was resuspended in 80 μL of MACS buffer (0.5% bovine serum albumin (BSA) and 2 mM EDTA), and 20 μL of CD45 magnetic beads, and incubated at 4 °C for 15 min. For CAF isolation, a positive selection kit with anti-fibroblast magnetic beads (Mylteni Biotech) was used and the cells were incubated with this reagent for 30 min at room temperature. The unbound beads were then washed out with 1 mL of MACS buffer and centrifuged at 300 xg for 5 min. The cells were re-suspended in 500 μL of MACS buffer and placed into the column located in a magnetic field that was pre-washed with 500 μL of MACS buffer. Following the addition of the cell solution, the column was washed 3 times with 500 μL of MACS buffer [25]. The unlabeled cells that passed through the column were fixed using 4% paraformaldehyde (PFA, Electron Microscopy Sciences) for 20 min and then cytospun onto a microscopy slide at 1000 rpm for 4 min. For CAF isolation, these cells were collected from the column, fixed and cytospun onto microscopy slides as well.

### CTC enumeration from PC patient blood samples

Slides were incubated with 100 μL of DPBS (Gibco) for 25 min. The cells were then permeabilized using 100 μL of 0.25% Triton-X solution (Sigma) for 15 min. Following permeabilization, the cells were immersed in 100 μL of 1% BSA for 1 h to block nonspecific interaction. The cells were stained with 100 μL of CD45-biotin (Clone HI30, 10 μg/mL, Biolegend) for 30 min. After washing, the cells were next stained with 100 μL of Streptavidin-Alexa Fluor 546 (10 μg/mL, Invitrogen), anti-Cytokeratin (Clone CAM5.2, 10 μg/mL, BD) in 0.05% Triton-X for 40 min. To identify CAFs, anti-Cytokeratin was replaced by anti-α-Smooth Muscle Actin (α-SMA, Clone 1A4, eBioscience). After each staining step, the cells were washed using 200 μL of DPBS three times. A drop of DAPI mounting media (Vectashield) was placed onto the cells, and the coverslip was placed and sealed with nail polish. Fluorescent micrographs were taken using CellSense software on an Olympus IX81 motorized inverted microscope. Cancer cells were identified using the following criteria: DAPI^+^, CD45^−^ and CK^+^. CAF enumeration utilized the following criteria: DAPI^+^, CD45^−^ and α-SMA^+^. To quantify the number of CTC clusters, each group of interacting cells involving more than 3 cells was identified as a CTC cluster [23].

### Preparation of TRAIL liposome therapeutic

Multilamellar liposomes were prepared using the thin film method as described previously [21]*.* Respective volumes were taken from the stock lipid solutions and gently mixed in a glass test tube and left under vacuum overnight. The lipid pellet was hydrated using 700 μL of liposome buffer (20 mM HEPES, 150 mM NaCl, pH 7.5). The multilamellar liposomes were generated using 10 cycles of freeze (2 min) and thaw (3 min). To prepare unilamellar liposomes, the liposomes were subjected to 10 extrusion cycles at 55 °C using two different size (200 nm and 100 nm) of polycarbonate membranes. For this study, we prepared two liposomal-treatment groups: control (liposomes that do not contain proteins on their surface) and TRAIL-liposomal therapy (E-selectin and TRAIL conjugated liposomes). Recombinant human E-selectin (R&D Systems) and recombinant human TRAIL (Enzo) were reconstituted to a final concentration of 500 μg/mL and 1 mg/mL and then stored at -80 °C. Freshly prepared liposomes were incubated with E-selectin and TRAIL to a final concentration of 71.43 nM and 250 nM for 15 min at 37 °C and then overnight at 4 °C on a rotator to prepare TRAIL-liposomal therapy [21]. The liposomes were characterized using a DLS-Malvern Zetasizer and a NanoSight NS300. This TRAIL-liposomal formulation has been previously used, characterized and validated in a previous study [21].

### Treatment of primary blood samples from PC patients under flow conditions

For the treatment group, 1.960 mL of PC patient blood was treated with 40 μL of functionalized liposomes (vehicle control or TRAIL therapy) and placed in a cone-and-plate viscometer that was pre-blocked with 1% BSA (Sigma) for 15 min. The blood was treated for 4 h at a physiological shear rate of 120 s^− 1^ to simulate the venous shear rate [26]. After 4 h, the treated blood aliquots were removed from the viscometer using HBSS buffer (Gibco) to wash out the blood and transferred to a new tube. The CTCs were isolated as previously described and placed in culture in a 6-multiwell plate using 3 mL of RPMI media (ATCC) overnight. After, the CTCs were collected from culture and stained with PI (BD) for 15 min. These cells were then fixed and cytospun onto microscope slides as indicated in the section *CTC enumeration from PC patient blood samples.*

### Statistical analysis

Patient samples at different time points were considered as biological repeats in the statistical analysis of single CTC enumeration, CAF enumeration, clustered CTC concentration, and the viability percentage of CTCs throughout the cancer treatment. Detailed information about the number of samples, the biological repeats and the statistical tests are explained in the respective figure legends. Student paired *t* test was used to compare two groups, whereas ANOVA tests where used to compare three or more groups for normally distributed data. For data that failed the normality test, Wilcoxon paired *t* and Friedman test were used to compare two and three groups, respectively. For multiple comparison, Turkey adjustments were applied and the adjusted *P* value was used. All tests were two-sided and at a level of significance =0.05. The statistical analyses were carried out in PRISM 6.0 for Mac OS X.

## Results

### CTC release by primary tumor surgical resection in PC patients

**Fig. 1:**
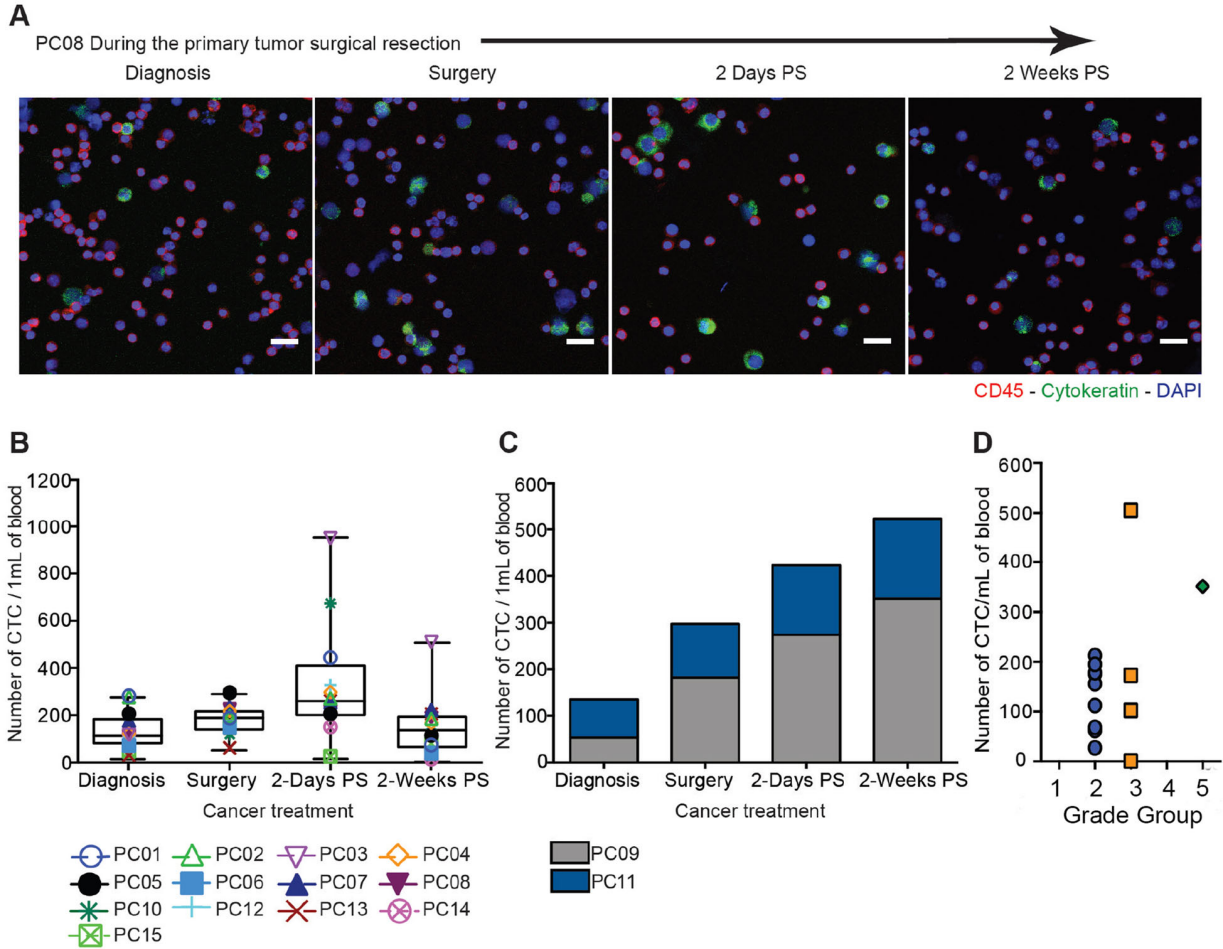
Fluctuations in the CTC counts induced by primary tumor resection **(a)** Immunofluorescent staining of CTCs isolated from one PC patient at different time points (red is CD45, green is cytokeratin and blue is DAPI) showing the increase in CTC level through cancer treatment. Scale bar represents 20 μm. **(b)** Box and whisker plot represents the CTC count in samples from PC patients at different time points: diagnosis, surgery, 2 days post-surgery and 2 weeks post-surgery. (mean ± SD, *n* = 48 samples from 13 patients). A slight increase of CTC (*P* = 0.0698) over surgical tumor removal was determined using one-way ANOVA. (**c)** A stacked column chart shows the CTC counts in PC patients who developed cancer recurrence at different time points (*n* = 8 samples from 2 patients). Significant increase (**P = 0.0030) in the number of CTCs following surgical resection of primary tumor was calculated using the correlation test. **(d)** CTC counts in PC patients at 2 weeks post-surgery compared with the grade of PC (*n* = 14 samples from 14 patients)

To investigate CTC mobilization induced by surgical resection of the primary PC tumor, 7.5 mL of blood was collected from 15 patients diagnosed with prostate adenocarcinoma at four time points throughout the cancer treatment process (Additional file 1 and Table 1). Blood from healthy donors was used as a negative control in which no CTCs were detected (Additional file 2). At the time of diagnosis, over 35 CTCs per mL were found in all PC patients participating in the study. Following surgical tumor resection, CTC level increased by more than two-fold compared to the baseline level at the time of diagnosis in 73% (11 of 15) of patients. After 2 weeks post-surgery, the CTC levels decreased to their initial level in 84% (11 of 13) of patients (Fig. 1A and B). These patients have not developed any cancer recurrence to date. However, in 13% (2 of 15) of patients, CTC levels increased gradually over time. These two patients with increasing CTC levels have experienced disease recurrence within months of the index procedure (Fig. 1C). Collectively, we found that a higher CTC level in the blood samples 2 weeks post-surgery correlates with the cancer stage, progression, and recurrence (Fig. 1D). Only two patients (13%) did not follow the correlation expected, which led us to hypothesize that there is likely a synergism of more than one factor involved.

**Table 1:** Summary of the clinical information of the 15 PC patients who participated in this study

Patient Characteristics	
Total, *N*	15
Age, yr, median (range)	62 (49–73)
PSA value at diagnosis, ng/mL, median (range)	5.9 (1.04–29.48)
Biopsy stage, *N* (%)	
1c	11 (73%)
1a	1 (7%)
2b	3 (20%)
Biopsy Gleason Grade Group, *N* (%)	
Grade Group 1 = 3 + 3	3 (20%)
Grade Group 2 = 3 + 4	6 (40%)
Grade Group 3 = 4 + 3	4 (27%)
Grade Group 4 = 4 + 4	2 (13%)
Surgery stage, *N* (%)	
2a	7 (47%)
3a	7 (47%)
3b	1 (6%)
Surgery Gleason score, *N* (%)	
Grade Group 2 = 3 + 4	10 (67%)
Grade Group 3 = 4 + 3	4 (27%)
Grade Group 5 = 4 + 5	1 (6%)
Nodes, *N* (%)	
Positive	1 (7%)
Negative	12 (80%)
Cannot access	3 (13%)
Surgical margins, *N* (%)	
Positive	3 (20%)
Negative	12 (80%)
Adjuvant therapy, *N* (%)	
ADT	2 (13%)
Radiation	1 (7%)
Cancer recurrence, *N* (%)	
Salvage treatment	1 (7%)
Biochemical recurrence	1 (7%)

### CTC mobilization observed in PC patients occurs in the form of CTC clusters

**Fig. 2:**
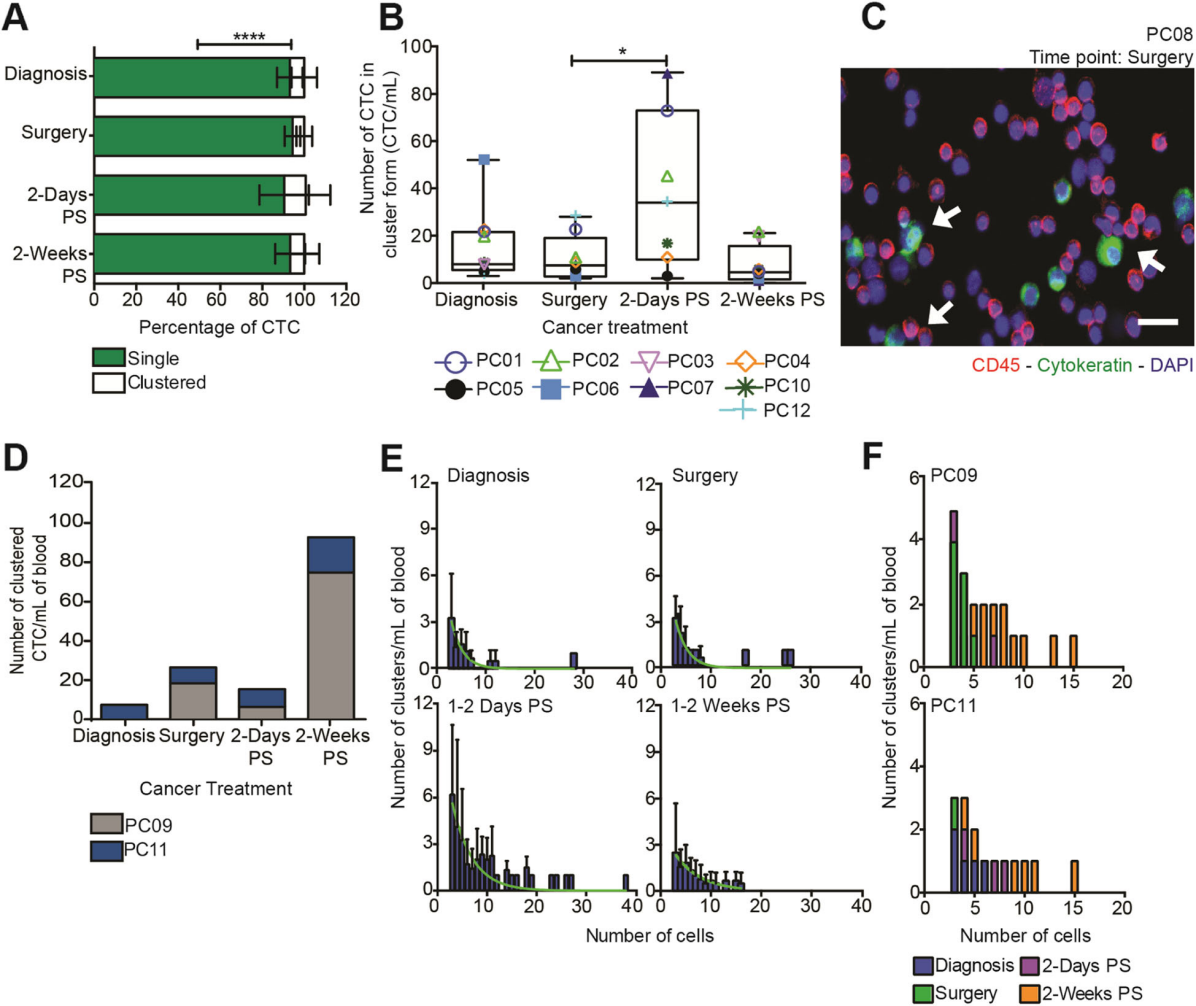
Variation in the CTC cluster numbers identified in PC patients undergoing primary tumor resection **(a)** A stacked column chart represents the classification of tumor cells found in PC patients as single and clustered CTCs before and after primary tumor resection (mean ± SD, *n* = 33 samples from 9 patients). The samples (Percentage of single CTC vs CTC in cluster form) were found to be significantly different (****P < 0.0001), according to a two-way ANOVA test. **(b)** Box and whisker plot of clustered CTC numbers at different time points during cancer treatment of PC patients. (mean ± SD, *n* = 33 samples from 9 patients). Significance (*P = 0.0469) was calculated using Wilcoxon paired t test. **(c)** Immunofluorescence images of CTC clusters found in PC patients (red is CD45, green is cytokeratin and blue is DAPI). Scale bar of 20 μm. (**d)** A stacked column chart represents the number of CTCs in cluster form before and after primary tumor resection in PC patients who developed cancer recurrence (*n* = 8 samples from 2 patients). **(e)** Column chart represents the size distribution of the CTC clusters identified in PC patients (mean ± SD, *n* = 33 samples from 9 patients). Superimposed curves represent a nonlinear Gaussian regression. **(f)** Column chart represents the size distribution of the CTC clusters identified in patients that developed cancer recurrence (*n* = 8 samples from 2 patients)

The number of CTCs in cluster form was evaluated, motivated by the documented advantage that CTC clusters hold over single cells in forming macro-metastases in distant organs (10). In all the patient blood samples, an average of 10% of all identified CTCs were found to be within CTC clusters (Fig. 2A). At the time of diagnosis, over 3 CTCs in the form of clusters per mL of blood were found in 91% (10 of 11) of patients. After surgical resection of the primary tumor, the number of CTCs in cluster form increased over two-fold compared to its initial level at the time of the diagnosis in 67% (6 of 9) of patients. Then, the clustered CTC concentration decreased to its initial level after 2 weeks post-surgery in 89% (8 of 9) of patients (Fig. 2B and C). All of these patients remained free from recurrence at the time of publication. On the other hand, 18% (2 of 11) of patients showed an increase in clustered CTC concentration coincident with primary tumor resection (Fig. 2D). These patients developed cancer recurrence within months after surgery. In addition to the clustered CTC level, we quantified the size distribution of CTC clusters in PC patients due to primary tumor resection. We found that PC patients exhibit mostly small CTC clusters composed of less than 15 cells per cluster at the time of diagnosis, whereas after the surgical resection of the primary tumor, PC patient blood contains an over two-fold greater number of small CTC clusters along with larger CTC clusters that included up to 40 cells per cluster. After 2 weeks post-surgery, we found that the size distribution attenuated back to baseline levels (Fig. 2E). However, in PC patients experiencing cancer recurrence, the size distribution increased in accordance with the primary tumor resection (Fig. 2F). This shows that CTC release due to surgery is not limited to single CTCs, but rather includes both single CTCs as well as CTC clusters. We also found that CTC cluster level correlates with the cancer stage, progression, and cancer recurrence.

### CAF enumeration in the bloodstream correlates with cancer recurrence in PC

**Fig. 3:**
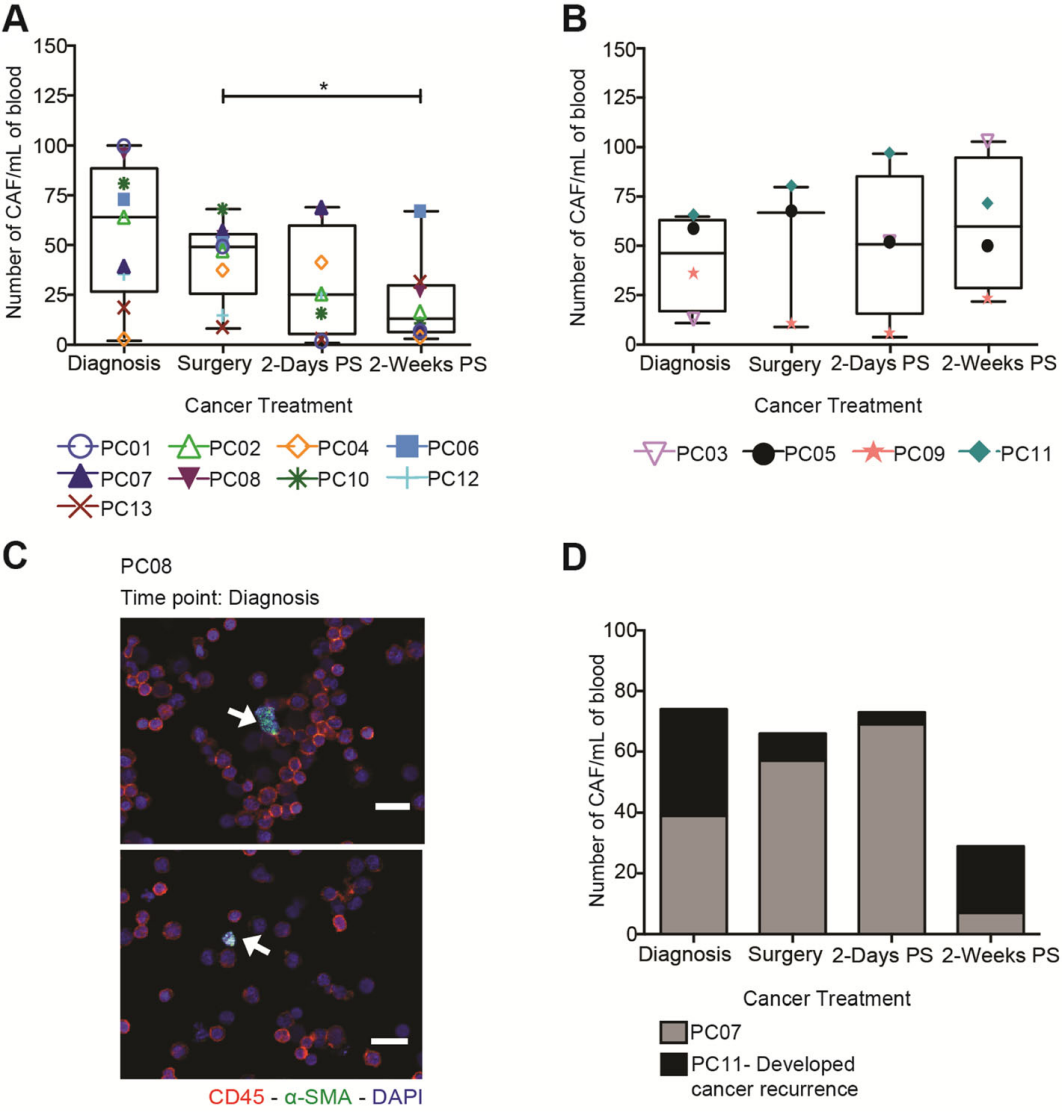
Fluctuations in the CAF counts induced by primary tumor resection in patients diagnosed with different stages of PC **(a)** Box and whisker plot represents the CAF counts from patients diagnosed with grade 1 prostate adenocarcinoma, before and after primary tumor resection (mean ± SD, *n* = 34 from 9 patients). There were significantly fewer (*P = 0.0422) CAFs after 2 weeks post-surgery compared with the baseline, as calculated using one-way ANOVA. **(b)** CAF counts from patients diagnosed with grade 2 and 4 prostate adenocarcinoma through primary tumor resection (*n* = 15 from 4 patients). There was no significant change found (*P* = 0.8020) in the CAF counts in these patients, as calculated using one-way ANOVA. **(c)** Immunofluorescence staining of CAFs isolated from PC patients (red is CD45, green is alpha-SMA and blue is DAPI). Scale bar represents 20 μm. **(d)** A stacked column chart represents CAF counts from patients with the same cancer stage and surgical resection outcomes (*n* = 8 samples from 2 patients)

Primary blood samples from 13 PC patients were used to evaluate CAF levels in blood at different time points following primary tumor resection. To determine the CAF concentration, fibroblasts were isolated via positive selection method from patient samples (Additional file 1C and D). Blood from healthy donors was used as a negative control in which no CAFs were detected. In addition, immunofluorescent staining of WPMY-1 (normal fibroblast cell line) and hTERT PF179 CAF (CAF cell line) were used as negative and positive controls (Additional file 3). At the time of diagnosis, blood collected from all of the PC patients contained over 2 CAFs per mL of blood. After 2 weeks following primary tumor resection, CAF counts decreased ~ 2-fold from its initial level at the time of diagnosis in 67% (6 of 9) PC patients diagnosed with grade 1 adenocarcinoma. These patients remain free from recurrence (Fig. 3A and C). However, in 31% (4 of 13) of patients diagnosed with advanced adenocarcinoma (grade 2 and 4), the CAF levels increased gradually despite the primary tumor removal which is considered the main source of CAFs in cancer patients (Fig. 3B). In the latter group of patients, two patients have developed cancer recurrence recently, one of which is identified as biochemical recurrence. Additionally, these patients also exhibited increased levels of clustered CTCs following primary tumor resection. To investigate the determining factor in promoting cancer recurrence, we compared two of the patients that have a similar clinical background. We found that the dominant factor associated with cancer recurrence was the increase in CAF and CTC cluster levels in the circulation (Fig. 3D). This observation suggests that CTC release occurs in CTC clusters, including stromal cells such as CAF, and that this can promote cancer progression and recurrence. However, this observation should be confirmed in other PC patients.

### TRAIL therapy killed CTCs within blood from patients undergoing primary tumor resection

**Fig. 4:**
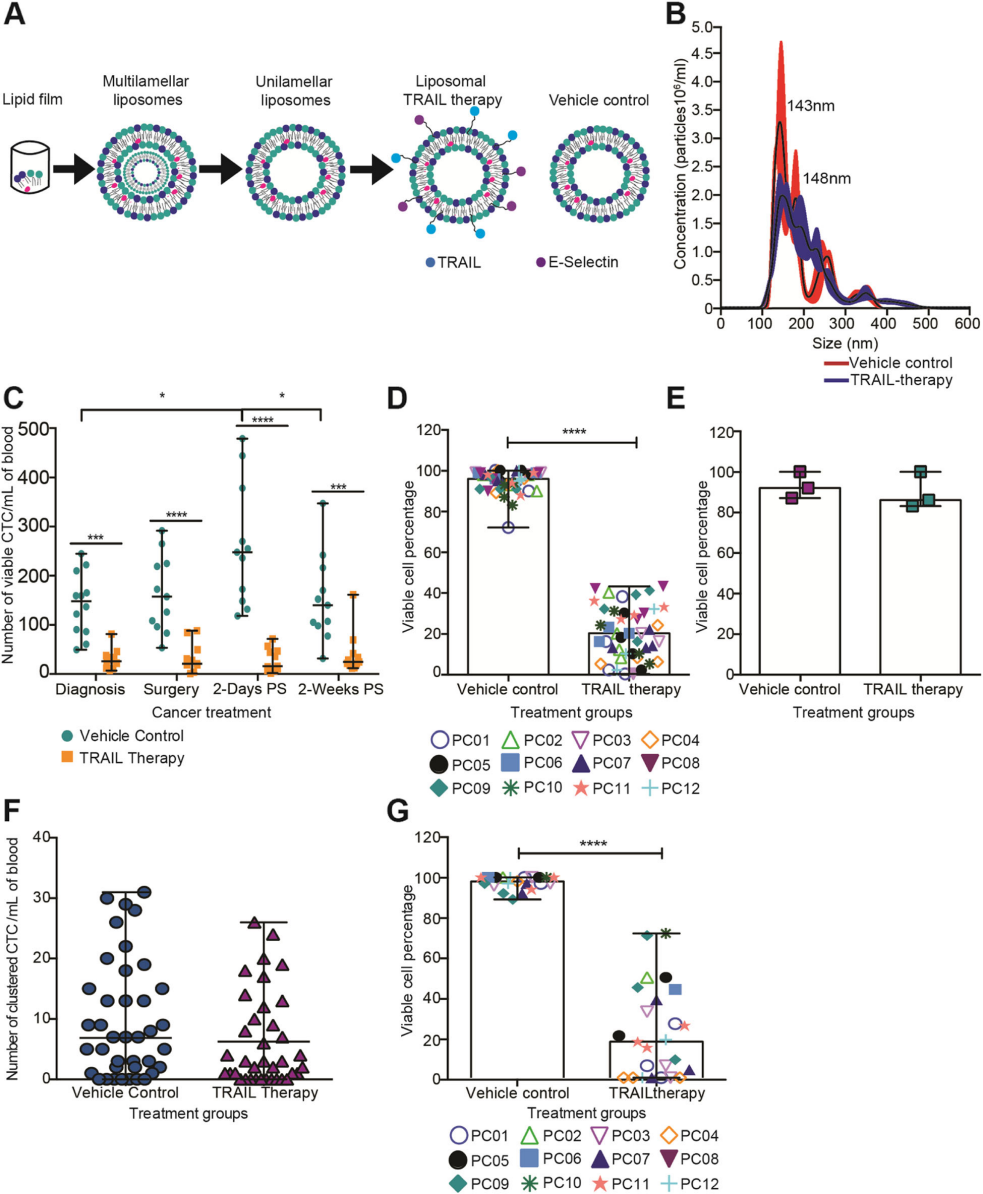
TRAIL liposomal therapy efficiently killed CTCs from PC patients **(a)** Experimental procedure to prepare the liposomal TRAIL therapy. **(b)** Size (diameter) distribution of nanoscale liposomes for non-conjugated (vehicle control) and conjugated (TRAIL therapy) formulations with TRAIL and E-selectin protein. The superimposed black curve represents the mean of three different runs (mean, *n* = 3 from 3 batch of liposomes prepared). **(c)** A scatter dot chart represents the viable CTC counts of samples treated with the vehicle control and TRAIL therapy (median ± range, *n* = 45 samples from 12 patients) through primary tumor resection. Significance with regard to the cancer treatment (*P < 0.0376) and to TRAIL therapy vs. vehicle control (**P < 0.0058, ***P = 0.0004 and ****P < 0.0001) was calculated in a two-way ANOVA test. **(d)** Column chart represents the viable CTC percentage treated with vehicle control and with the TRAIL therapy (mean ± SD, *n* = 45 samples from 12 patients). There was a significant reduction (****P < 0.0001) in cell viability percentage, as calculated in a Wilcoxon paired *t* test. **(e)** Column chart represents the viable CTC percentage of samples treated with PBS and with soluble TRAIL (*n* = 3 samples from 1 patient). Non-significance (*P =* 0.1994) was calculated in a paired *t* test. **(f)** Scatter dot plot that represents the number of CTCs in the form of clusters in samples treated with the vehicle control and with the TRAIL therapy (mean ± range, *n* = 41 samples from 11 patients). Non-significance (*P =* 0.0826) was calculated for a Wilcoxon paired *t* test. **(g)** Column chart represents the percentage of viable CTCs in clustered form in samples treated with the vehicle control and TRAIL therapy (mean ± SD, *n* = 25 samples from 10 patients). There was a significant (****P < 0.0001) decrease in viability percentage in clustered CTC with the TRAIL therapy compared with the vehicle control, as calculated in a Wilcoxon paired *t* test

To evaluate the efficacy of our previously developed TRAIL therapy for killing CTCs in blood samples from PC patients, we treated primary patient blood with TRAIL- and E-selectin-coated liposomes as described previously (20, 22, 23) (Fig. 4A). In this study, the vehicle control had a size of 117.67 nm ± 0.13 with a polydispersity index (PDI) of 0.09 ± 0.02 while our TRAIL liposomes had a final size of 130.60 nm ± 1.40 with a PDI value of 0.17 ± 0.01, which confirms the presence of proteins on the liposome surface (Additional file 4). With regard to the liposome concentration, the vehicle control contained 6.40 ± 0.39 (× 10^12^) particles/mL while the liposomal TRAIL therapy contained 5.90 ± 0.26 (× 10^12^) particles/mL (Fig. 4B). After completing the characterization of our nanomedicine construct, a 290 ng/mL dose of the vehicle control and the TRAIL therapy was used to treat CTCs in 2 mL of whole blood isolated from PC patients in a cone-and-plate viscometer for 4 h at room temperature (Additional file 5 S1B). Patient blood treated with TRAIL/E-selectin liposomes showed an over two-fold decrease in CTC count compared to the control group treated with empty vehicles, in all PC patients at the time of diagnosis (Fig. 4C). Importantly, after surgical tumor resection TRAIL therapy reduced the CTC level in PC patients consistently, regardless of the magnitude of CTC increase induced by the surgical procedure. In summary, this therapeutic agent significantly killed over 75% of CTCs in PC patient blood samples (Fig. 4D). To determine the efficacy of the TRAIL therapy to target CTCs, three patient samples were treated with soluble TRAIL at the same concentration that was incorporated in the liposomal formulation (290 ng/mL) under the same experimental conditions. Soluble TRAIL did not kill CTCs in blood samples from PC patients, as expected due to lack of E-selectin which creates a cell-membrane tethered delivery of TRAIL to CTCs (Fig. 4E). However, since we found a considerable concentration of clustered CTCs in PC patients, the remaining question to address was whether this therapy holds the same efficacy to kill tumor cells in flowing blood when they are assembled into clusters instead of single cells.

### TRAIL-therapy reduces clustered CTC viability under physiological levels of fluid shear stress

E-selectin is a natural cell adhesion receptor expressed by endothelial cells to support rolling adhesion of leukocytes in the post capillary venules [27]. However, experimental evidence demonstrates that cancer cells also express E-selectin ligands, supporting the idea that these cancer cells may use a similar mechanism to leukocytes for extravasating at inflammation sites, allowing CTCs to enter into foreign tissue during the metastatic process [28, 29]. Previous work by our lab has shown that E-selectin/TRAIL liposomes specifically attach to leukocytes due to E-selectin/ligand interactions, and engage with cancer cells via frequent blood cell collisions. Considering this, we evaluated the ability of this TRAIL therapy to induce the formation of CTC clusters via E-selectin interaction. As expected, it was determined that the TRAIL therapy did not induce the formation of CTC clusters under physiological shear conditions. These results show non-significant increases in clustered CTC concentration in flowing blood aliquots treated with the TRAIL therapy, in comparison to aliquots treated with the vehicle control (Fig. 4F). Interestingly, we found that the TRAIL therapy reduced the viability of clustered CTCs by killing over 75% of CTCs assembled in clusters (Fig. 4G).

## Discussion

Radical prostatectomy is the first line of treatment for localized prostate cancer followed, in some cases, by radiotherapy and ADT. In this study, we showed that surgery triggered mobilization of tumor cells from the primary tumor into the circulation and that CTC release occurs in the form of both single CTCs and CTC clusters. Moreover, the single CTC and clustered CTC levels were found to normalize after 2 weeks post-surgery in most patients. Although prostatectomy in most patients resulted in cancer remission due to complete removal of the tumor with negative surgical margins and no tumor recurrence after 1 year of follow up, the increase in CTC level after surgery was observed in a small cohort of patients. In this study, we identified that 2 out of 15 patients experienced cancer recurrence within 2 months after prostatectomy. These patients had an increase in single CTC and clustered CTC levels at or after surgery and the levels did not normalize after 2 weeks. These findings are consistent with previously published results suggesting that the presence of CTC clusters correlates with a higher probability of cancer recurrence and worse prognosis [30]. Based on the literature, the CTC clusters include stromal cells such as CAF, which not only promote their survival but the colonization of distant tissues as well [14].

In this study, we detected a decrease in CAF level during cancer treatment in patients diagnosed with lower grade prostate adenocarcinoma. However, the opposite behavior was observed in patients diagnosed with higher grade prostate adenocarcinoma that exhibited rapid cancer recurrence. A significant difference was observed when two patients that were diagnosed at the same cancer stage and had similar resection outcomes were compared. It was found that the determinant factor in cancer recurrence is higher CAF and CTC cluster levels in the circulation. Thus, for the first time we found that in PC, CAFs may play an important role in promoting CTC survival under high hemodynamic forces by promoting the growth of tumor cells locally, and eventually causing cancer recurrence. Collectively, we found that CAF levels may correlate with a worse prognosis, disease progression, and cancer recurrence in PC. However, this observation was found in a small cohort of patients, therefore additional studies are encouraged to confirm this observation and to elucidate the cellular mechanisms involved.

**Fig. 5:**
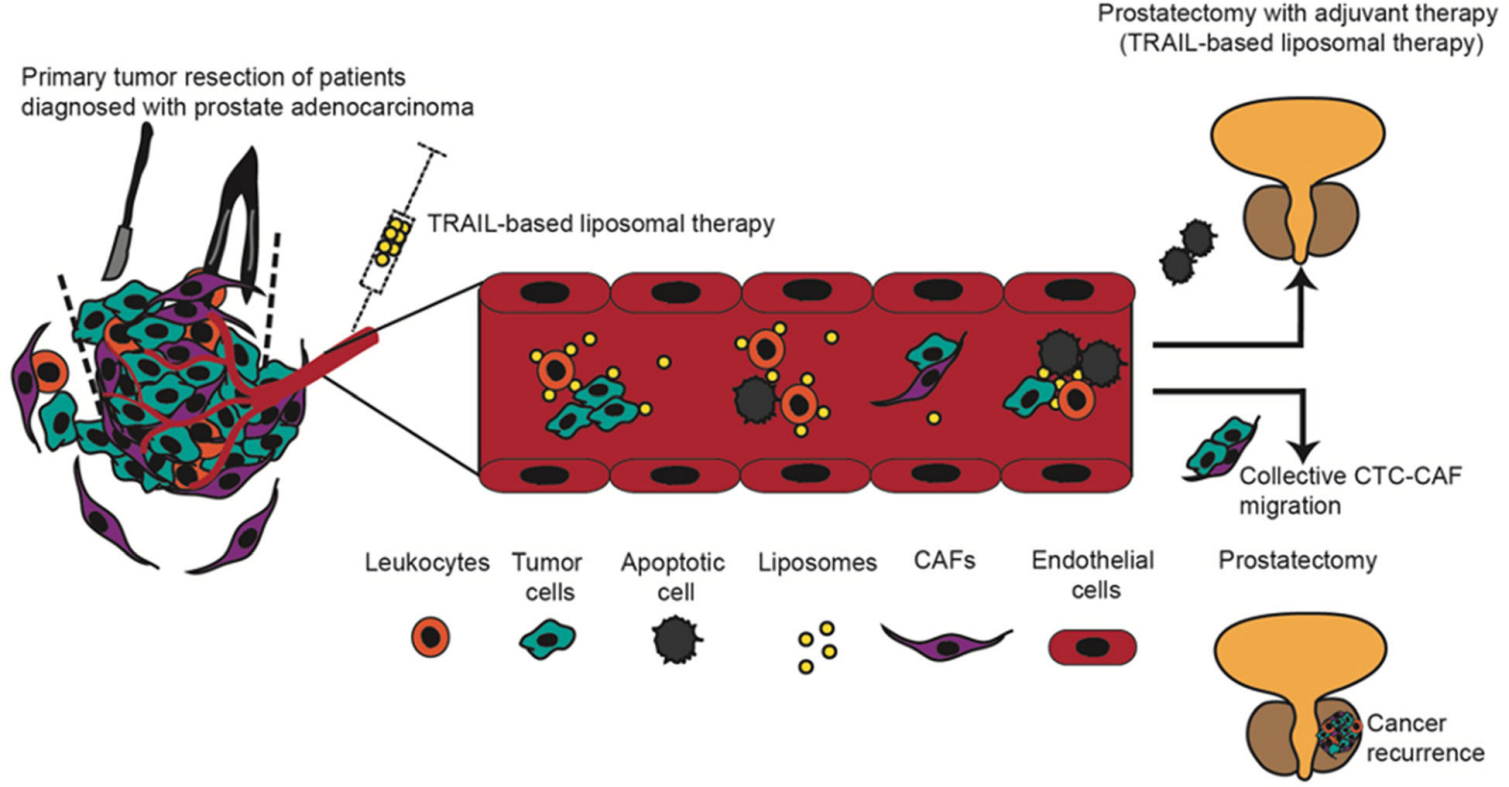
Possible mechanism of how CTC-CAF cluster migration promotes cancer recurrence and the proposed use of TRAIL therapy as an adjuvant therapy to prevent this cancer recurrence during surgery. During the primary tumor resection, CTCs are released into the bloodstream due to the disruption of the tumor microenvironment. The CTCs can migrate as clusters along with other CTCs or stromal cells such as CAFs to facilitate the survival and growth in distant tissues. After radical prostatectomy, the CTC cluster and CAF numbers in blood correlate with cancer recurrence in PC patients. Thus, we propose the use of TRAIL therapy as an adjuvant therapy that could be administered during the peri-operative window to neutralize newly released CTCs caused by surgical procedures. Ex vivo, this approach killed over 75% single CTCs and clustered CTCs, respectively. We believe that the eradication of most of the single and clustered CTCs in the bloodstream could prevent cancer recurrence and progression

Circulating tumor cells and stromal cell mobilization due to primary tumor resection lends support to the idea that adjuvant therapy to reduce CTC clusters or CTC or CAF levels in the bloodstream, may prevent or delay cancer recurrence and metastasis. Studies have reported that the administration of chemotherapy after primary tumor resection improves overall survival in cancer patients [31, 32]. However, studies have suggested some concern related to the use of chemotherapy post-surgery due to adverse effects on wound healing and immune response [33]. On the other hand, reports have indicated the use of ADT in combination with prostatectomy to decrease the probability of cancer recurrence [34]. Despite the reduction of cancer recurrence, for high risk PC, tumor cells can develop resistance to therapy and recur at local sites or spread to distant tissue [35]. In this study, we tested our previously developed TRAIL liposomal therapy and determined that this approach killed over 75% of single CTCs and clustered CTCs, regardless of the increase in CTC levels that occurred after primary tumor resection. In accordance with the present results, previous studies have demonstrated that this liposomal therapy reduces an estimated 84% of CTCs in an orthotopic prostate murine model, and this reduction was enough to prevent the formation of metastases in distant tissues such as lung, liver, kidney and spleen in comparison with mice treated with the vehicle control. In addition, the TRAIL therapy reduced the primary tumor burden [22]. Therefore, we believe that a 75% reduction of CTC due to TRAIL therapy could be sufficient to prevent or delay cancer recurrence. Comparing TRAIL-based liposomal therapy with chemotherapy and ADT, liposomal therapy efficiently targets and eliminates CTCs in the bloodstream within only a few hours of exposure without harming host cells, including endothelial cells and leukocytes as was demonstrated in previous studies [21]. Indeed, TRAIL liposomal therapy is a promising adjuvant therapy that could be used in conjunction with prostatectomy to effectively neutralize CTC release and potentially prevent cancer recurrence (Fig. 5).

The current study has one limitation that deserves mention. The blood samples were shipped from the University of Rochester Medical Center to Vanderbilt University overnight, where all samples were processed within 48 h. Reports suggest that CTC levels do not fluctuate significantly in blood samples processed within 24, 48 and 72 h from collection [24]. If there is a variation in the CTC numbers due to the viability of the CTCs fluctuating over the time from blood draw, this may introduce some systematic or random error to our study. Nevertheless, all of the samples were processed and analyzed using the same shipping and experimental conditions, which should reduce bias from collection.

## Conclusion

In conclusion, we have documented that CTC mobilization occurs during or within 2 days of surgery in PC patients. We identified that this CTC release occurs partially in the form of CTC clusters, which may include stromal cells such as CAFs. This suggests the use of CTC cluster and CAF counts as a predictive biomarker for possible cancer recurrence after surgical tumor resection. Successful elimination of CTCs with TRAIL-liposomal therapy provides proof of concept to its application as adjuvant therapy in PC patients undergoing surgery with curative intent.

## Supplementary Information

**Additional file 1 **Experimental design used in this study to process and analyze the blood samples of cancer patients**. (A)** Blood samples were collected at the University of Rochester Medical Center and shipped overnight to Vanderbilt University to be processed within 48 h. Four blood samples were collected from 15 patients at different time points: diagnosis, surgery, 2 days post-surgery and 2 weeks post-surgery. **(B)** Two aliquots of 1.96 mL were treated with 40 μL of vehicle control and TRAIL therapy for a total volume of 2 mL and loaded into a cone-and-plate viscometer. The samples were sheared for 4 h at room temperature. **(C)** The CTC isolation was carried out using a two-step process: buffy coat isolation, and then CTC purification via negative selection of CD45-positive cells (leukocytes). For CAF isolation, we performed a positive selection of fibroblast cells (CAF). **(D)** Immunofluorescent staining was used to identify CTCs, viable CTCs after treatment, and CAFs in the blood samples from cancer patients.

**Additional file 2.** No CTCs were found in blood samples collected from healthy donors. (A) Immunofluorescent staining of CTCs from blood samples of healthy donors isolated using the same protocol used to isolate CTCs from prostate cancer patients (red is CD45, green is cytokeratin and blue is DAPI). Scale bar is 20 μm.

**Additional file 3.** Validation of isolated fibroblasts from prostate cancer patients as CAFs. (A) Immunofluorescent staining of two fibroblast cell lines obtained from ATCC and used as positive and negative controls (green is α-SMA and blue is DAPI). Scale bar is 20 μm. WPMY-1 is a myo-fibroblast cell isolated from the peripheral area of the prostate. hTERT PF179 CAF is a fibroblast cell line derived from prostate cancer stroma. Staining with α-SMA (biomarker to identify CAFs from normal fibroblast cells) confirmed that the α-SMA positive cells found in the prostate cancer patients are CAFs instead of normal fibroblasts. (B) Immunofluorescent staining of CAFs isolated from a healthy donor (green is α-SMA, red is CD45 and blue is DAPI). Scale bar is 20 μm. No CAFs were found in blood samples from healthy donors (*n* = 3 from 5 healthy donors).

**Additional file 4.** Characterization of prepared liposomes using a Malvern Zetasizer and NanoSight NS300 with the following parameters presented: size (diameter), polydispersity index (PDI) and particle concentration (particles/mL).

## Data Availability

The datasets used and analyzed in this study are available from the corresponding author upon reasonable request.

## Abbreviations

PC: Prostate cancer
CTC: Circulating tumor cell
CAF: Cancer-associated fibroblast
TRAIL: TNF-related apoptosis inducing ligand
RP: Radical prostatectomy
XT: Radiation
ADT: Androgen deprivation therapy
UNT: Untreated
BSA: Bovine serum albumin
PDI: Polydispersity index
