# Supplementary material for: TRAIL-coated leukocytes to kill circulating tumor cells in the flowing blood from prostate cancer patients
Source: BMC Cancer. 2021 Aug 6;21:898. doi: 10.1186/s12885-021-08589-8 (PMC8343922; doi:10.1186/s12885-021-08589-8)
Supplement: Supplementary file 1 — Additional file 1 Experimental design used in this study to process and analyze the blood samples of cancer patients. (A) Blood samples were collected at the University of Rochester Medical Center and shipped overnight to Vanderbilt University to be processed within 48 h. Four blood samples were collected from 15 patients at different time points: diagnosis, surgery, 2 days post-surgery and 2 weeks post-surgery. (B) Two aliquots of 1.96 mL were treated with 40 μL of vehicle control and TRAIL therapy for a total volume of 2 mL and loaded into a cone-and-plate viscometer. The samples were sheared for 4 h at room temperature. (C) The CTC isolation was carried out using a two-step process: buffy coat isolation, and then CTC purification via negative selection of CD45-positive cells (leukocytes). For CAF isolation, we performed a positive selection of fibroblast cells (CAF). (D) Immunofluorescent staining was used to identify CTCs, viable CTCs after treatment, and CAFs in the blood samples from cancer patients. [file 12885_2021_8589_MOESM1_ESM.docx]

**
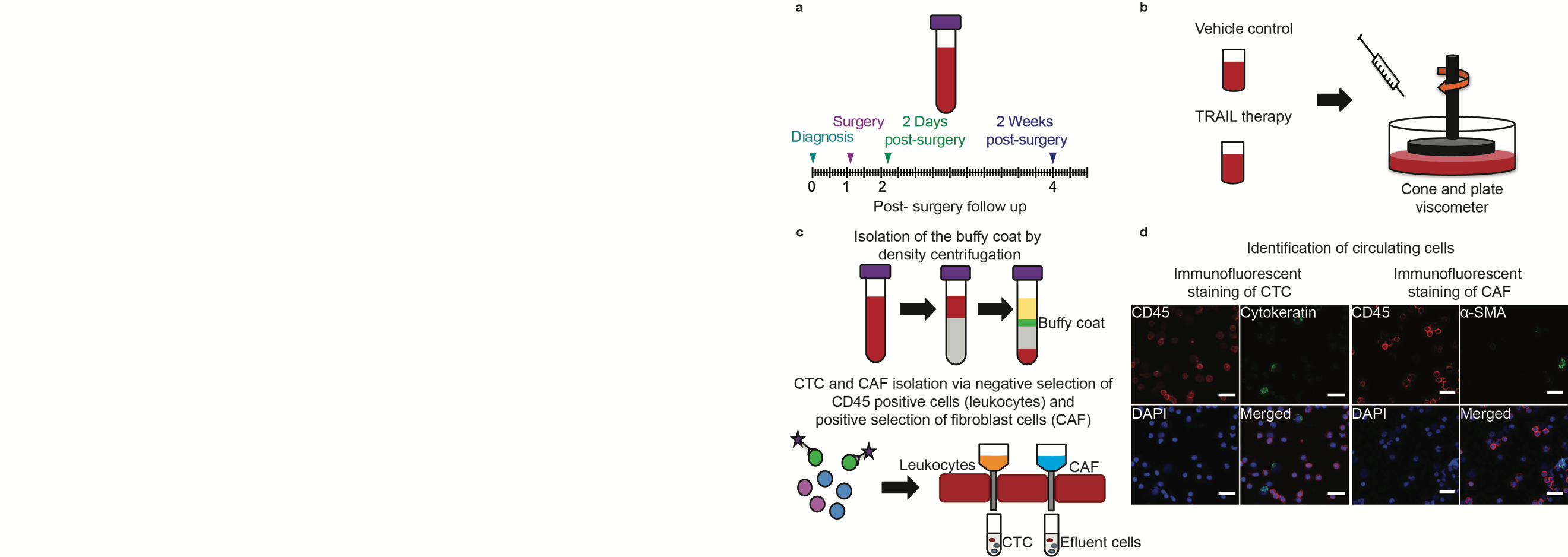
**

Additional file 1. Experimental design used in this study to process and analyze the blood samples of cancer patients**.** **(A)** Blood samples were collected at the University of Rochester Medical Center and shipped overnight to Vanderbilt University to be processed within 48 hr. Four blood samples were collected from 15 patients at different time points: diagnosis, surgery, 2 days post-surgery and 2 weeks post-surgery. **(B)** Two aliquots of 1.96 mL were treated with 40 µL of vehicle control and TRAIL therapy for a total volume of 2 mL and loaded into a cone-and-plate viscometer. The samples were sheared for 4 hr at room temperature. **(C)** The CTC isolation was carried out using a two-step process: buffy coat isolation, and then CTC purification via negative selection of CD45-positive cells (leukocytes). For CAF isolation, we performed a positive selection of fibroblast cells (CAF). **(D)** Immunofluorescent staining was used to identify CTCs, viable CTCs after treatment, and CAFs in the blood samples from cancer patients.
