# Supplementary material for: TRAIL-coated leukocytes to kill circulating tumor cells in the flowing blood from prostate cancer patients
Source: BMC Cancer. 2021 Aug 6;21:898. doi: 10.1186/s12885-021-08589-8 (PMC8343922; doi:10.1186/s12885-021-08589-8)
Supplement: Supplementary file 2 — Additional file 2. No CTCs were found in blood samples collected from healthy donors. (A) Immunofluorescent staining of CTCs from blood samples of healthy donors isolated using the same protocol used to isolate CTCs from prostate cancer patients (red is CD45, green is cytokeratin and blue is DAPI). Scale bar is 20 μm. [file 12885_2021_8589_MOESM2_ESM.docx]

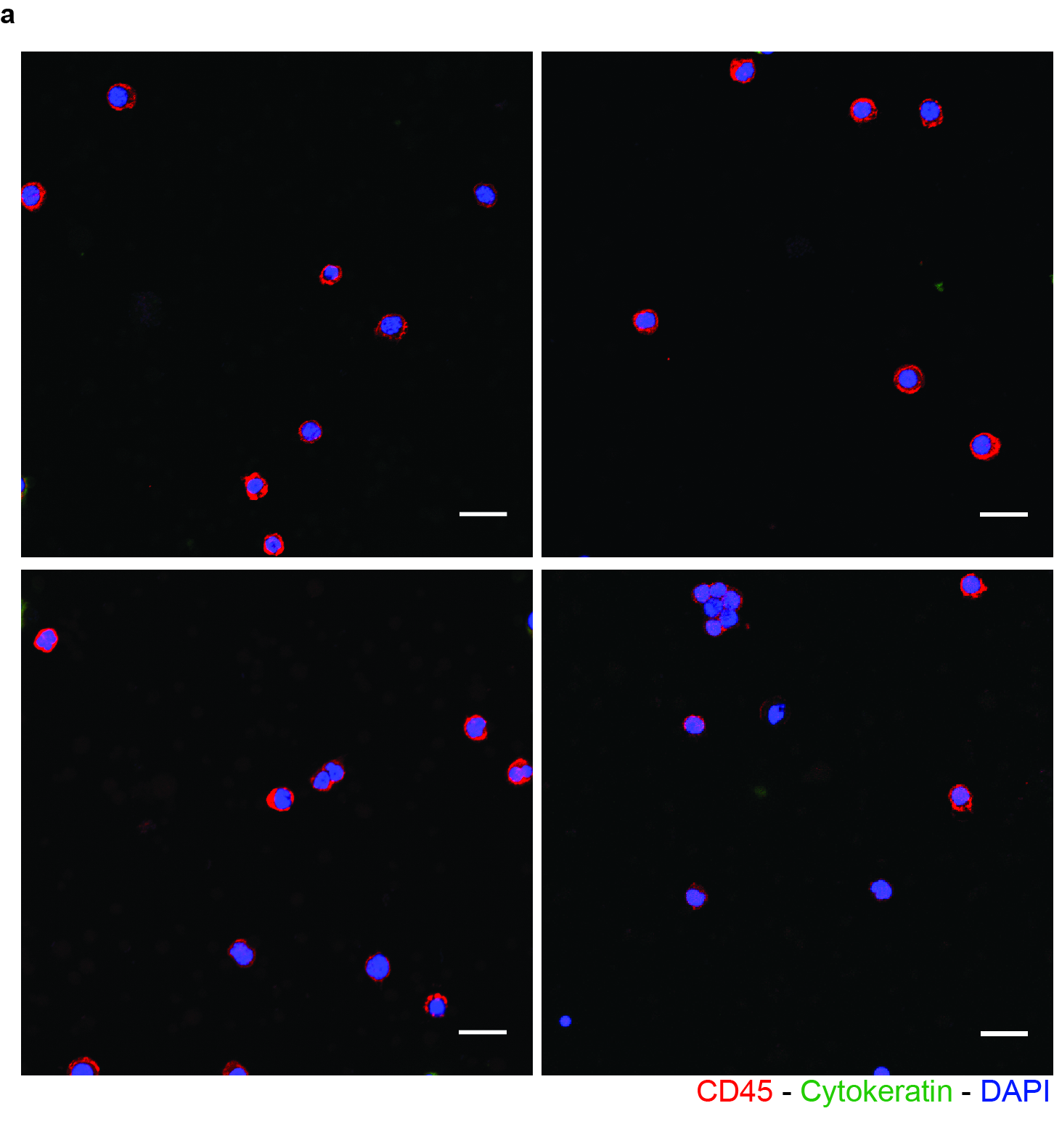


**Additional file 2: No CTCs were found in blood samples collected from healthy donors. (A)** Immunofluorescent staining of CTCs from blood samples of healthy donors isolated using the same protocol used to isolated CTCs from prostate cancer patients (red is CD45, green is Cytokeratin and blue is DAPI). Scale bar is 20 µm.
