# Supplementary material for: TRAIL-coated leukocytes to kill circulating tumor cells in the flowing blood from prostate cancer patients
Source: BMC Cancer. 2021 Aug 6;21:898. doi: 10.1186/s12885-021-08589-8 (PMC8343922; doi:10.1186/s12885-021-08589-8)
Supplement: Supplementary file 3 — Additional file 3. Validation of isolated fibroblasts from prostate cancer patients as CAFs. (A) Immunofluorescent staining of two fibroblast cell lines obtained from ATCC and used as positive and negative controls (green is α-SMA and blue is DAPI). Scale bar is 20 μm. WPMY-1 is a myo-fibroblast cell isolated from the peripheral area of the prostate. hTERT PF179 CAF is a fibroblast cell line derived from prostate cancer stroma. Staining with α-SMA (biomarker to identify CAFs from normal fibroblast cells) confirmed that the α-SMA positive cells found in the prostate cancer patients are CAFs instead of normal fibroblasts. (B) Immunofluorescent staining of CAFs isolated from a healthy donor (green is α-SMA, red is CD45 and blue is DAPI). Scale bar is 20 μm. No CAFs were found in blood samples from healthy donors (n = 3 from 5 healthy donors). [file 12885_2021_8589_MOESM3_ESM.docx]

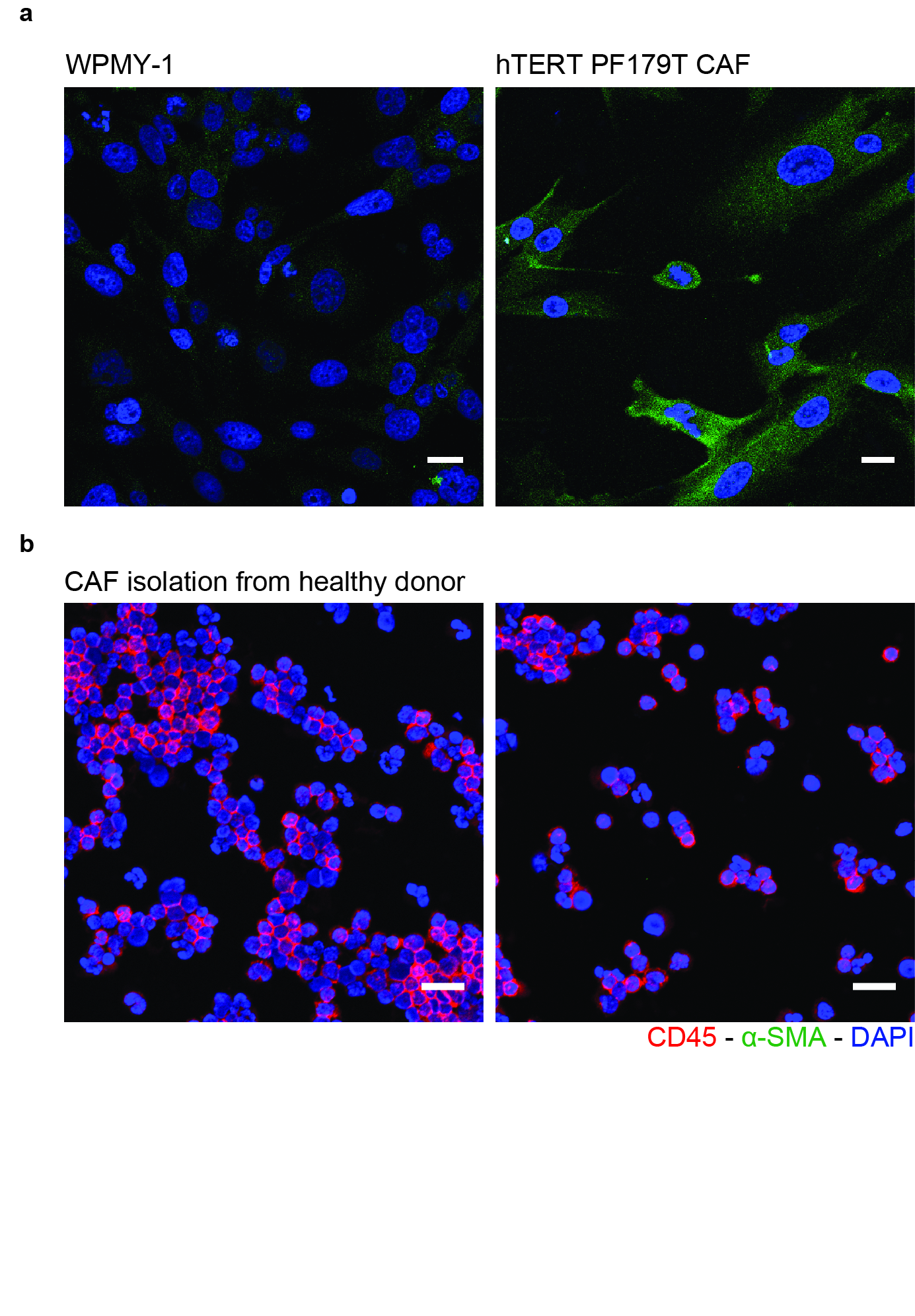


**Additional file 3: Validation of isolated fibroblasts from prostate cancer patients as CAFs. (A)** Immunofluorescent staining of two fibroblast cell lines obtained from ATCC and used as positive and negative controls (green is α-SMA and blue is DAPI). Scale bar is 20 µm. WPMY-1 is a myo-fribloblast cell isolated from the peripheral area of the prostate. hTERT PF179 CAF is a fibroblast cell line derived from prostate cancer stroma. Staining with α-SMA (biomarker to identify CAFs from normal fibroblast cells) confirmed that the α-SMA positive cells found in the prostate cancer patients are CAFs instead of normal fibroblast. **(B)** Immunofluorescent staining of CAFs isolated from a healthy donor (green is α-SMA, red is CD45 and blue is DAPI). Scale bar is 20 µm. No CAFs were found in blood samples from healthy donors (*n*=3 from 5 healthy donors).
