# Supplementary material for: TRAIL-coated leukocytes to kill circulating tumor cells in the flowing blood from prostate cancer patients
Source: BMC Cancer. 2021 Aug 6;21:898. doi: 10.1186/s12885-021-08589-8 (PMC8343922; doi:10.1186/s12885-021-08589-8)
Supplement: Supplementary file 4 — Additional file 4. Characterization of prepared liposomes using a Malvern Zetasizer and NanoSight NS300 with the following parameters presented: size (diameter), polydispersity index (PDI) and particle concentration (particles/mL). [file 12885_2021_8589_MOESM4_ESM.docx]

**Additional file 4: Characterization of prepared liposomes using Zetasizer and NanoSight with the following parameters presented: size (diameter), polydispersity index (PDI) and particle concentration (particles/mL)**

| Sample | Diameter (nm) | PDI value | Concentration (particles x10^12^/mL) |
| --- | --- | --- | --- |
| Vehicle control | 117.66±0.13 | 0.09±0.02 | 1.60±0.39 |
| TRAIL therapy | 130.60±1.40 | 0.17±0.01 | 1.48±0.26 |
